# Correspondence

**Published:** 1867-05

**Authors:** 


### Correspondence.

### Correction by the Northwestern Christian Advocate.

66 Washington St., Chicago, April 23, 1867.

J. F. Miner, M. D.:

Dear Sir:—I have received your March and April numbers, and have been much gratified by your kind notice of our article on "Criminal Abortion." We will continue the war in some shape. In your April number you speak of the *Northern Christian Advocate.* Our *Advocate* family is large and our paper is called the *Northwestern.* The *Northern* is published at Auburn, N. Y., and your misprint will mislead most of your readers.

We, as editors, fully agree with you in your denunciation of a species of advertisements. These we cannot control, as they are in the hands of our *publishers.* We wish we could entirely expurgate them.

With kindest regards,

I am, yours truly,

A. Edwards.

---

Peekskill, March 25, 1867.

Dr. Miner—My Dear Sir:

I send you the inclosed Table, relating to Senators of United States, prepared by my friend, J. M. Toner, M. D. You will probably think it worth publishing, together with his comments in the accompanying *Medical Register.* I think the facts worthy of preservation in a durable form.

In haste, very truly,

Yours, etc.,

C. A. Lee.

### Senators of the 1st Session, 39th Congress.

The following table showing the physical characteristics of the members of the U. S. Senate, was prepared in July, 1866, by Frank Cowan, Esq., with great care, from inquiry and actual measurements made by himself. The table, with an able introductory article, on the subject of the figures and the Etymologies of the names of the Senators alphabetically arranged, was published in *Sutton's Reporter*, Washington, January 14, 1867:

*Table of the Physical Characteristics of the Members of the Senate. Prepared* 1*st*—10*th July*, 1866.

| NAMES. | HEIGHT Feet | HEIGHT Inches | WEIGHT Lbs. | CHEST Inches. | HEAD Inches. | BIRTH |
|---|---|---|---|---|---|---|
| Anthony | 5 | 11½ | 192 | 41 | 22 | 1 April, 1815 |
| Brown | 5 | 7¼ | 119 | 33½ | 22¼ | 28 May, 1826 |
| Buckalew | 5 | 9⅝ | 129 | 34 | 22½ | 28 Dec., 1821 |
| Chandler | 6 | 1⅜ | 208 | 41¼ | 22¾ | 10 Dec., 1813 |
| Clark | 6 | ¾ | 154 | 35¼ | 23 | 24 Oct., 1809 |
| Conness | 5 | 6⅝ | 166 | 39 | 23 | 6 Oct., 1822 |
| Cowan | 6 | 3¼ | 186 | 40 | 22 | 19 Sept., 1815 |
| Cragin | 5 | 9⅜ | 177 | 39¼ | 23½ | 3 Feb., 1821 |
| Creswell | 5 | 8⅜ | 201 | 41½ | 23¼ | 18 Nov., 1828 |
| Davis | 5 | 5⅜ | 127½ | 36 | 23 | 10 Sept., 1801 |
| Dixon | 5 | 10½ | 175 | 40 | 23½ | 5 Aug., 1814 |
| Doolittle | 5 | 10⅛ | 200 | 41 | 23¼ | 3 Jan., 1815 |
| Edmunds | 6 | ⅞ | 143 | 35½ | 22¾ | 1 Feb., 1828 |
| Fessenden | 5 | 10 | 130 | 33½ | 22½ | 16 Oct., 1806 |
| Foster | 5 | 11½ | 140 | 33 | 23 | 22 Nov., 1806 |
| Grimes | 5 | 11⅜ | 161 | 37¾ | 24 | 20 Oct., 1816 |
| Guthrie | 6 | 2 | 220½ | 42½ | 22¾ | 5 Dec., 1792 |
| Harris | 6 | 1 | 219 | 42½ | 23 | 31 May, 1802 |
| Henderson | 6 | 1 | 132½ | 35½ | 22½ | 16 Nov., 1826 |
| Hendricks | 5 | 9⅜ | 175 | 39¾ | 23½ | 7 Sept., 1819 |
| Howard | 5 | 9½ | 206 | 42¾ | 23½ | 10 July, 1805 |
| Howe | 6 | ⅞ | 163½ | 38 | 23¼ | 24 Feb., 1816 |
| Johnson | 5 | 6⅞ | 170½ | 38½ | 23½ | 21 May, 1796 |
| Kirkwood | 5 | 10¾ | 172 | 37½ | 22½ | 20 Dec., 1813 |
| Lane, of Indiana | 5 | 11½ | 142 | 34 | 21 | 24 Feb., 1811 |
| Lane, of Kansas | 5 | 11 | 140 | 38½ | 23 | 22 June, 1814 |
| McDougall | 5 | 6⅜ | 134 | 37 | 23 | 9 Nov., 1817 |
| Morgan | 6 | 1⅜ | 202½ | 42 | 23 | 8 Feb., 1811 |
| Morrill | 5 | 8⅞ | 152 | 36½ | 22¾ | 3 May, 1813 |
| Nesmith | 5 | 9⅝ | 200 | 41½ | 24 | 23 July, 1820 |
| Norton | 5 | 7⅝ | 148 | 36 | 22 | 12 April, 1829 |
| Nye | 5 | 9½ | 191½ | 42 | 23¼ | 10 June, 1815 |
| Poland | 5 | 9⅞ | 163 | 38¼ | 23¾ | 1 Nov., 1815 |
| Pomeroy | 5 | 9 | 232 | 44 | 24 | 3 Jan., 1816 |
| Ramsey | 5 | 10¾ | 211½ | 41 | 23 | 8 Sept., 1815 |
| Riddle | 5 | 9½ | 117 | 32¾ | 23 | 26 Nov., 1817 |
| Saulsbury | 5 | 11¾ | 183 | 38½ | 23 | 2 June, 1820 |
| Sherman | 6 | 2¼ | 157 | 35 | 23 | 10 May, 1823 |
| Sprague | 5 | 8⅝ | 145 | 34 | 22¼ | 12 Sept., 1830 |
| Stewart | 6 | 1⅜ | 187 | 40 | 23 | 9 Aug., 1827 |
| Sumner | 6 | 3⅛ | 204½ | 39¾ | 23 | 6 Jan., 1811 |
| Trumbull | 5 | 10½ | 150 | 35¾ | 22 | 12 Oct., 1813 |
| Van Winkle | 5 | 7¾ | 234 | 43 | 24 | 7 Sept., 1808 |
| Wade | 5 | 8¾ | 185 | 41½ | 23 | 27 Oct., 1800 |
| Willey | 6 | 1 | 162½ | 38 | 22½ | 18 Oct., 1811 |
| Williams | 6 | 1 | 178 | 40½ | 22¾ | 26 March, 1823 |
| Wilson | 5 | 10 | 180½ | 40¼ | 22¼ | 16 Feb., 1812 |
| Wright | ... | ... | ... | ... | ... | 1791 |
| Yates | 5 | 9½ | 153 | 36 | 23 | 18 Jan., 1817 |
| Averages | 5 | 10½ | 171¼ | 38⅓ | 22⅚ | *Yrs.* 51 *Months.* 11 *Days.* 14 |

**Note.**—The above measurements of height were taken with boots on—measurements of chest over vest but under the coat.

It appears from this physical survey of the personnel of the U. S. Senate that the tallest member "is Mr. Cowan, the shortest, Mr. Davis, while fourteen of the forty, are six feet in height; the heaviest, Mr. Van Winkle, the lightest, Mr. Riddle, while twelve weigh two hundred or more. The Senator with the largest chest, Mr. Pomeroy; the smallest, Mr. Riddle; while nineteen have chests measuring in circumference forty inches or more; the possessors of the largest heads are Messrs. Grimes, Nesmith, Pomeroy and Van Winkle, the smallest, Mr. Lane, of Indiana, while thirty have heads measuring in circumference twenty-three inches or more; the oldest, Mr. Wright, the youngest, Mr. Sprague, and thirty-one have lived for more than half a century."

The foregoing table shows that in all the points observed our Senators exceed the average of mankind in all parts of the world as well as the average of our own country. As legislative bodies in their selection bear a certain relationship to the masses of the people so do their averages bear a relationship one to the other, but whether or not these averages are proportionate has not yet been ascertained. That is whether or not the higher and more select the body the more striking the relationship between it and the people from which it is selected. The investigation started by Mr. F. Cowan is novel, and as yet has not been carried far enough nor contrasted with similar legislative bodies in other countries to make the deductions which more extensive investigations in this direction will enable us to do. Is it true that because the average of physical development of our Senators is greater than the average of their countrymen, that, therefore they have greater mental and moral power?

That lineage and race stamp mental and moral characteristics and capacities as well as physical peculiarities upon individuals is a well recognized fact in the science of ethnology.

The lineage or nationalities deduced from the etymology of the names of the Senators differs slightly from those given by themselves, but the variation is so trifling as not to impair the law.

| | English | Scotch | Welsh | German | Dutch | French | Irish |
|---|---|---|---|---|---|---|---|
| Deduced from Etymology of names. | 29 | 8 | 4 | 3 | 2 | 2 | 1 |
| Ancestry as given by the Senators.* | 23 | 8 | 3¾ | ¾ | ¾ | 2 | 4¾ |

* Six not given.

The preponderance of Senators of English descent is very great. When the proportion of nationalities represented in our whole population is known the significance of these figures will be increased.

From the interesting and carefully prepared table of Mr. Frank Cowan it appears that the average "age of the Senators was on the 1st of July, 1866, 51 years, 11 months and 14 days."

The mean life-time or average duration of life in America as inferred from elaborate examinations of records of population and mortality in Massachusetts, and of other States from d ta worked up by Mr. E. B. Elliott, M. A., delegate from the American Staistical Association to the late International Statistical Congress at Berlin, Prussia, 1863, is about forty years; this does not differ essentially from the average in England and Wales, and in France, but is somewhat higher than in Prussia and certain other European countries.

The mean age of a generation in America considered as *stationary*, as derived from the same records that is between 32 and 33 years, and the mean future life-time of such generation is also between 32 and 33 years.

The mean age, and also the mean future life-time of a generation of people in England are each nearly the same—that is, between 32 and 33 years.

During the working period of life, say from the age of 15 to 45, and also in early infancy, the rate of mortality appears to be somewhat greater in the United States than in England. During the remaining period—that is, childhood and advance years—it is less here. The rate of mortality among infants on the continent appears in general to exceed the American rates.

The average age of soldiers in the volunteer forces of the United States during the late civil war was from 23 to 26 years. That army, as would probably prove the case with all volunteer armies, having been supplied and recruited mainly from the young.

Mr. Elliott has shown that the mortality of soldiers at specified ages of life in the army diminishes rapidly with advanced age, and in accordanee with a very simple law—that is, that the differences of the numbers at different ages of life diminish very nearly by a geometrical progression.

The average ages of the soldiers in the British army (not recruits) serving at home in 1860, was 23 years. The average age of European troops in India for ten years, 1847 to 1857, was 23½ years.

The average age of soldiers, officers and men serving in the Prussian army in 1861, was 23½ years.

The average height of Senators of the Thirty-ninth Congress, according to the measurements of Mr. Cowan, is about 70½ inches, (or five feet ten and a half inches;) which is about two and a half inches (or 3⅝ per cent.) in excess of the average height of mankind.

The average height of men in general is probably about sixty-eight inches. The height of recruits to the late volunteer forces of the United States, according to Mr. Elliott's data, was from sixty-seven to sixty-eight inches. That of recruits to the regular army of the United States, for a series of years prior to the late war, appears to have been nearer sixty-nine (68.8) inches.

From the report of the Provost Marshal General of the United States, we find that in the examination of 237,391, the average height barefooted was found to be nearly sixty-seven and a half inches.

While in forty-seven nationalities collected in the same report, the average of 343,764 examined, the average height was found to be sixty-six and three-quarter inches, nearly.

That of recruits to the British army in the years 1860 and 1861, was sixty-six and one-half inches. That of British soldiers, (not recruits, but in general older and consequently somewhat taller than recruits,) in 1846, was sixty-eight and one-half inches. That of French conscripts for a long series of years, sixty-five and one-fifth (65-21) United States inches. That of French soldiers sixty-five and three-fourths (65.77) United States inches. From that it appears that the French soldier is somewhat shorter than either the English or American; the two latter being nearly of the same height.

The mean variation of the height of the American volunteers from the average height was about two inches; being the same in excess as in defect.

The average measurement about the chest of the Senators is 38⅓ inches, which exceeds somewhat that of men generally. The circumference of the chest (taken under the coat and vest) of United States volunteers of the late army of the Potomac, was about 35 inches. The mean variation of the measurements from the average circumference of these soldiers was one and two-thirds inches; and the same in excess as in defect.

The three States presenting the tallest average men, according to the Provost Marshal's report, Minnesota, sixty-seven inches and ninety-one hundredths; Kansas, sixty-seven and thirty-five hundredths; Kentucky, sixty-seven and thirty-four hundredths. The shortest, the District of Columbia, sixty-six inches; West Virginia, sixty-five inches and twenty-eight hundredths; and New Hampshire, sixty-five inches and twelve hundredths.

The average chest measurements as given in the Provost Marshal General's Report, thirty-five inches and sixty-one hundredths on respiration, and thirty-three and eleven hundredths of an inch on expiration. The same measurements collected from forty-seven nationalities found in the same report is found to be—on respiration, 35.59; on expiration, 33.12.

The three States giving the largest average chest measurements on inspiration were Kentucky, thirty-six inches and thirty-seven hundredths; Maine, thirty-six inches and five hundredths; and Illinois, thirty-six inches and nineteen hundredths. The smallest, Rhode Island, (33.00) inches; New Hampshire, (34.32) inches; and Massachusetts, (34.34) inches.

The weight of the Senators was about 171¼ pounds avoirdupois; that of soldiers in army of Potomac having been about 147½ pounds, which probably differs but little from, although, perhaps, somewhat less than the average weight of Americans generally.

The weight of recruits, already mentioned, to the British army in 1860 and 1861, was nearly one hundred and thirty pounds, (129½) which is somewhat less than that of the American soldiers, but their ages were also less, consequently some such difference might be expected.

The average circumference of the heads of the Senators, taken a little lower than the hat is usually worn, is about twenty-two and five-sixths (22.83) inches; that of the U. S. volunteers above mentioned having been about twenty-two and one-fifth (22.2) inches. The measurements of the U. S. soldiers were taken about the frontal eminence, and the greatest projection of the occiput—largest measurement—the process not differing essentially from that adopted with the Senators. The excess of the measurement in case of Senators was about two-thirds of an inch.

---

*To the Editor of the Buffalo Medical and Surgical Journal:*

That the inhalation of oxygen gas is destined to become an important healing agent, may be inferred from the success which has already attended it. In sickness a preponderence of oxygen is necessary to decompose the carbonized matter in the blood, and to excite new action in the diseased parts. Over fifty years ago Dr. Beddoes began to treat diseases of the lungs by the employment of various gases to be inhaled from a bladder; and of twenty-two cases of asthenia treated by him by the inhalation of oxygen, ten were cured and nine relieved. M. Demarquay, who has devoted much attention to the use of oxygen inhalation in medicine, says, in speaking of its therapeutic indications, that, in the early stage of phthisis, where there is no fever, and no fear of exciting local action, when the patient is becoming emaciated, and emaciation is increased by persistent dyspepsia, oxygen may have a salutary influence in modifying the state of the constitution and sustaining the organism. In senile gangrene, as long as the circulation continues in the artery of the foot, oxgen is, according to the observations of MM. Langier, Demarquay and Maurice Reynaud, the only remedy which in advanced cases affords a chance of recovery. The inhalation of oxygen is beneficial not only in pulmonary and bronchial affections, but also after typhus, typhoid and other low types of fevers, wherein an impoverished condition of the blood exists, lacking in the vitalizing principles of oxygenation. Oxygen renders incontestable service in essentiel anæmia. It is especially indicated in that form of chlorosis of young girls which is characterized by obstinate anorexia; in the anæmia of convalescents, and in the anæmia, often severe, of newly-delivered females. The inhalation of oxygen is also successful in anæmia arising from hemorrhage or from fatigue, and is also a very energetic remedy in the debility produced by prolonged suppuration; it stimulates the appetite, sustains the powers of the patient, and enables him to attain to recovery. In diabetes, under the influence of oxygen inhalation, the quantity of sugar contained in the urine is remarkably diminished. (*Gaz. Med. de Paris*, 14 *April*, 1866.)

The primary action of oxygen inhalation is prompt, while its secondary or more remote effects are permanent and highly salutary. The invigoration is continuous and persistent without subsequent depression, as is the case with most other stimulants. I have used with great success protoxide of nitrogen, with the addition of two-thirds of pure oxygen gas, the natural affinity of nitrogen and oxygen favoring such a combination. This can be medicated, since owing to its rapid absorption by the blood, rem edies are made to permeate the system much sooner and more effectively than when applied through the medium of the stomach and lacteals. The gas should be generated at a nearly uniform heat between 400° and 410°. The gas should be *strained* through at least four or five carefully perforated glass vessels holding from one-half to two gallons each, so that every portion may come in contact with the washing fluid. The gas, for preservation, should be confined in properly constructed metallic vessels over water.

During the past year I have administered this combination to over fifty persons with remarkable success. The Rev. S. I. D. came to my office from an adjoining city, suffering from phthisis. Upon examination I found tubercles upon his left lung extending as low as the third rib. It was with difficulty that he could walk the short distance from his lodgings to my office. The inhalation of oxygen gas arrested the deposit of tubercles. In nine weeks he had gained six pounds in weight and was able to walk three or four miles every day. He has now resumed his duties as pastor, and considers himself a well man. G. S. P. had suffered for fifteen years with catarrh; he was partially deaf, was unable to smell, and his eyes were very weak. He has now been inhaling oxygin six weeks. His deafness is entirely relieved; he has regained his sense of smell, and I have no doubt but that he will be entirely cured. In scrofula its effects are magical. I have also given it for asthma, bronchitis, rheumatism, neuralgia, paralysis, dyspepsia, nervous debility and mercurial diseases with the greatest success. CHAS. H. S. DAVIS.

Meriden, Conn.

---

MERIDEN, Conn., 4th mo., 30, 1867.

Dr. J. F. Miner:

Dear Sir:—My attention was first attracted to the inhalation of oxygen by reading Birch's work "On the Therapeutic Action of Oxygen with cases proving its singular efficacy in various Intractable Diseases;" London, 1837. Davy's "Chemical and Philosophical Researches," 1793, and a small work "On the Inhalation of Gases and Medicated Vapors, by W. Abbotts Smith, M. D., M. R. C. P."

I know that ignorant pretenders have made a specialty of the inhalation of oxygen and other gases, but I am confident from the experience that I have had with it that there is great remedial virtue in it. I notice that Dr. Sprague, in the *Boston Medical and Surgical Journal* for May 17, 1866, and Dr. McLain in the *New Orleans Medical and Surgical Journal* for March, 1867, have recommended the use of nitrous oxide in the treatment of disease.

I never put this foremost as a cure-all, but in old chronic cases where I have tried everything else, I then try the effects of oxygen, and always with benefit. I have now under treatment a lady who has suffered for the past six years with catarrh, having lost entirely her sense of smell. I have given her about two gallons of oxygen every day for about three weeks, and she has now partially recovered her sense of smell and has very little discharge from the nose.

I should be pleased to write an article on the subject for publiction, giving my own experience. I should be pleased to inform any regular practitioner the whole *modus operandi* of the manufacture of the gas, and other information in regard to its inhalation.

Yours, respectfully,

CHAS. H. S. DAVIS, M. D.

